# Kallikrein directly interacts with and activates Factor IX, resulting in thrombin generation and fibrin formation independent of Factor XI

**DOI:** 10.1073/pnas.2014810118

**Published:** 2021-01-28

**Authors:** Katherine J. Kearney, Juliet Butler, Olga M. Posada, Clare Wilson, Samantha Heal, Majid Ali, Lewis Hardy, Josefin Ahnström, David Gailani, Richard Foster, Emma Hethershaw, Colin Longstaff, Helen Philippou

**Affiliations:** ^a^Leeds Institute of Cardiovascular and Metabolic Medicine, University of Leeds, LS2 9JT Leeds, United Kingdom;; ^b^Faculty of Medicine, Department of Immunology and Inflammation, Imperial College London, Hammersmith Campus, W12 0NN London, United Kingdom;; ^c^Division of Hematology/Oncology, The Vanderbilt Clinic, Vanderbilt University, Nashville, TN 37232;; ^d^School of Chemistry, University of Leeds, LS2 9JT Leeds, United Kingdom;; ^e^Division of Biotherapeutics, National Institute for Biological Standards and Control, Potters Bar, Hertfordshire, EN6 3QG, United Kingdom

**Keywords:** plasma kallikrein, prekallikrein, Factor IX, intrinsic pathway, Factor XII

## Abstract

Kallikrein (PKa), generated by activation of its precursor prekallikrein (PK), plays a role in the contact activation phase of coagulation and functions in the kallikrein-kinin system to generate bradykinin. The general dogma has been that the contribution of PKa to the coagulation cascade is dependent on its action on FXII. Recently this dogma has been challenged by studies in human plasma showing thrombin generation due to PKa activity on FIX and also by murine studies showing formation of FIXa-antithrombin complexes in FXI deficient mice. In this study, we demonstrate high-affinity binding interactions between PK(a) and FIX(a) using surface plasmon resonance and show that these interactions are likely to occur under physiological conditions. Furthermore, we directly demonstrate dose- and time-dependent cleavage of FIX by PKa in a purified system by sodium dodecyl sulfate-polyacrylamide gel electrophoresis analysis and chromogenic assays. By using normal pooled plasma and a range of coagulation factor-deficient plasmas, we show that this action of PKa on FIX not only results in thrombin generation, but also promotes fibrin formation in the absence of FXII or FXI. Comparison of the kinetics of either FXIa- or PKa-induced activation of FIX suggest that PKa could be a significant physiological activator of FIX. Our data indicate that the coagulation cascade needs to be redefined to indicate that PKa can directly activate FIX. The circumstances that drive PKa substrate specificity remain to be determined.

## Significance

Prekallikrein (PK) is a zymogen that is converted to kallikrein (PKa) by factor (F)XIIa. PK and FXII reciprocally activate each other; the resulting FXIIa initiates activation of the coagulation system via the cleavage of FXI to FXIa, which then activates FIX. This manuscript describes a novel high-affinity binding interaction between FIX(a) and PK(a) and reports that PKa can dose- and time-dependently activate FIX to generate FIXa, resulting in thrombin generation and clot formation independent of FXIa. Characterization of the kinetics of FIX activation reveal that PKa is a more significant activator of FIX than previously considered. This work highlights a new amendment to the coagulation cascade where PKa can directly activate FIX.

Hemostasis is a highly adaptive process that controls blood fluidity and rapidly induces hemostatic plug formation after vascular injury to stop or limit bleeding (1, 2). Traditionally, the coagulation cascade has been recognized as the result of triggering of the tissue factor (TF) (extrinsic) pathway or following contact activation, leading to initiation of the intrinsic pathway (3, 4). The extrinsic pathway is triggered on damage to a vessel wall and exposure of TF, which promotes activation of Factor (F) VII to FVIIa. TF/FVIIa form a complex which activates FX to FXa, allowing FXa to associate with cofactor FVa to form the prothrombinase complex, which serves to convert prothrombin into thrombin. Thrombin then cleaves fibrinogen to form fibrin, which polymerizes to form a network of fibrin fibers. The intrinsic pathway of coagulation is initiated by activation of FXII following exposure to negatively charged surfaces or molecules such as RNA, DNA, misfolded proteins, and polyphosphates (polyP) (5–10). Both FXII and prekallikrein (PK) of the intrinsic pathway possess zymogenic proteolytic activity that can initiate reciprocal activation to FXIIa and kallikrein (PKa), respectively. While this can occur in solution, the reaction is enhanced on a surface (11, 12). In the presence of a surface reciprocal activation of FXII and PK is amplified through the cofactor activity of high molecular weight kininogen (HK) (13, 14). The FXIIa generated stimulates the intrinsic pathway of coagulation by activating FXI, which then converts FIX to FIXa, and FIXa activation of FX to FXa, at which point the intrinsic and extrinsic pathways converge.

In addition to its role in the intrinsic pathway of coagulation, PKa also functions in the kallikrein-kinin system by cleaving HK, releasing the vasoactive peptide bradykinin (BK) (15). PK deficiency is associated with prolonged activated partial thromboplastin time (aPTT) (16, 17), but clinically, most PK-deficient patients have no hemostatic defect (18, 19). Murine in vivo PK deficiency is recognized to cause delayed arterial thrombosis induced by application of ferric chloride (20), while selective reduction of PK in vivo using antisense oligonucleotides in mice results in reduced thrombus formation without a bleeding defect (21). A study by Stavrou et al. (22) demonstrated that reduction of thrombosis in PK-deficient mice could be attributed to a mechanism other than reduced contact activation. The authors concluded that in the absence of PK, decreased production of BK in the PK-deficient mice was associated with reduced expression of the bradykinin B2 receptor (B2R), which led to a compensatory overexpression of Mas receptor. This was associated with increased plasma prostacyclin, which resulted in up-regulation of vascular transcription factors sirtuin-1 (Sirt1) and Kruppel-like factor 4 (KLF4) and a reduction in vascular TF (22). Within the coagulation cascade, the key substrate of PKa was considered to be FXII, leading to FXIIa activation of FXI and ultimately to thrombin generation. Early studies performed in a purified system identified activation of FIX by PKa (23) but reported that FXIa was more efficient at cleaving FIX (24). Sun and Gailani (25) demonstrated cleavage of FIX by PKa in a purified system, and Puy et al. (26) reported that FXIIa activated with polyP could reduce clotting time in FXI-deficient plasma by a mechanism dependent on FIX but independent of FXI . More recently, Visser et al. (27) demonstrated indirectly (using a specific PKa inhibitor in FXI-deficient plasma) that PKa contributes to FXIa-independent activation of FIX, as determined by measuring FIXa-antithrombin and FXa-antithrombin complexes, thrombin generation, and aPTT prolongation. Furthermore, Visser et al. showed elevated FIXa-antithrombin complex levels when mice deficient in FXI were challenged with ellagic acid and long-chain polyP, which were reduced in the presence of a specific PKa inhibitor. Noubouossie et al. (28) also indirectly demonstrated the ability of PKa to activate FIX.

The objectives of the current study were to directly demonstrate an interaction between PK(a) and FIX(a), to characterize the activation of FIX by PKa in purified and plasma-based systems, and to determine whether PKa activation of FIX leads to fibrin formation.

## Results

### PKa-Induced Thrombin Generation and Fibrin Formation Occur in the Absence of FXII and FXI.

**Fig. 1.**
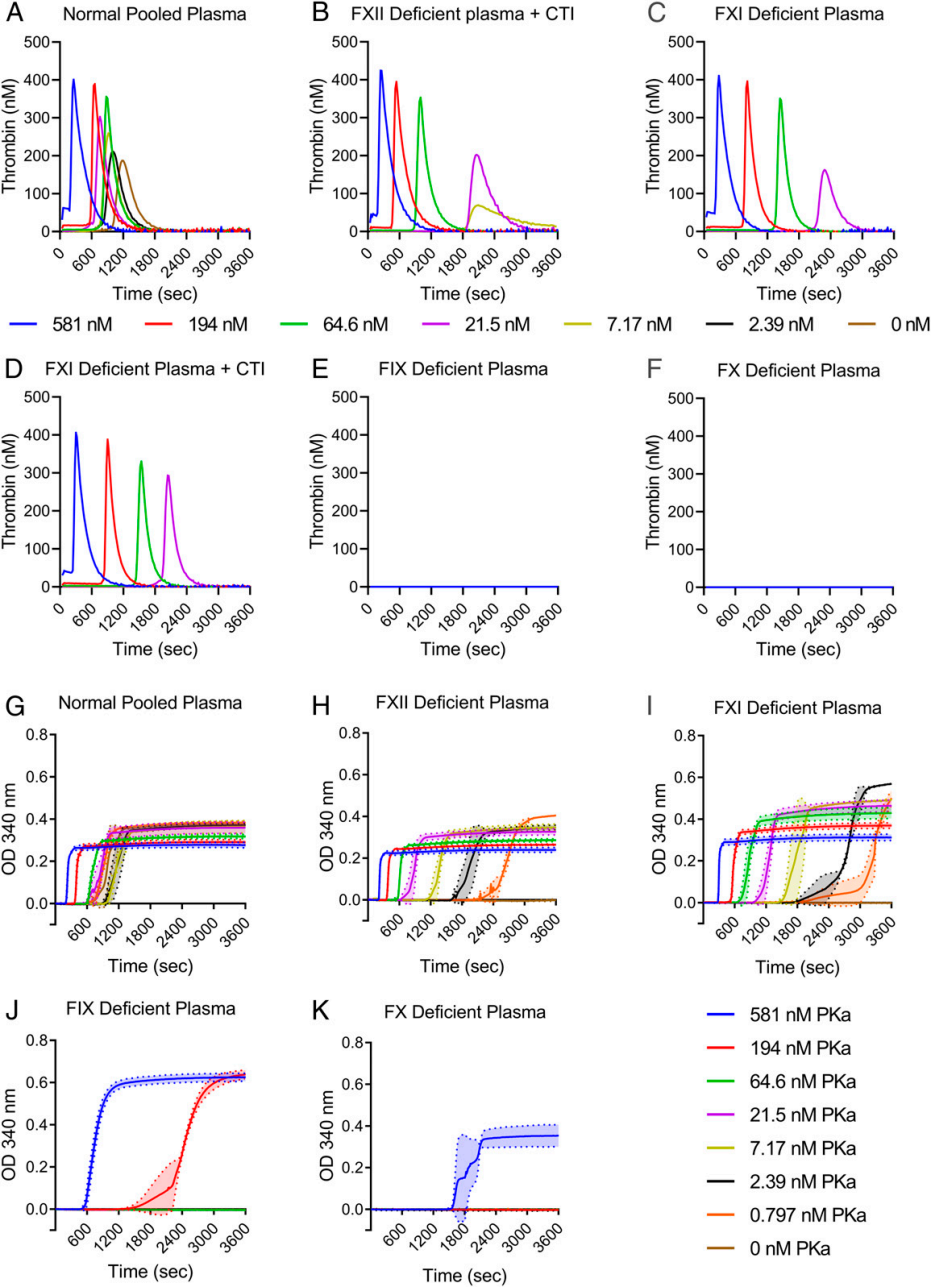
PKa can initiate thrombin generation and fibrin formation independently of FXII. Plasmas deficient in proteins of the clotting cascade were assessed for thrombin generation and fibrin formation when initiated with PKa (0.797 to 581 nM). (*A*–*F*) Thrombin generation performed after recalcification with 16.7 mM CaCl_2_ in the presence of 10 μM PLs. Thrombin generation in NPP (*A*), FXII-deficient plasma with CTI (*B*), FXI-deficient plasma (*C*), FXI-deficient plasma with CTI (*D*), FIX-deficient plasma (*E*), and FX-deficient plasma (*F*). Thrombograms show mean thrombin generated. *n* = 3. (*G*–*K*) Turbidimetric experiments were performed in plasma in the presence of 10 mM CaCl_2_ and 10 μM PLs. Fibrin clot formation over time was measured by the change in absorbance at 340 nm in NPP (*G*), FXII-deficient plasma (*H*), FXI-deficient plasma (*I*), FIX-deficient plasma (*J*), and FX-deficient plasma (*K*). Data are presented as mean ± SD, *n* = 3.

To assess whether PKa could initiate thrombin generation independently of its FXII-activating activity, thrombin generation was measured in FXII-deficient plasma spiked with increasing concentrations of PKa in the presence of phospholipids (PLs) and CaCl_2_. The highest concentration of PKa used in these assays was 581 nM, to reflect the plasma concentration of its precursor PK (15). Compared to normal pooled plasma (NPP) (Fig. 1*A*), thrombin generation occurred but was less efficient in FXII-deficient plasma (Fig. 1*B*). The addition of corn trypsin inhibitor (CTI) indicated that no residual FXII was contributing to PKa-induced thrombin generation (Fig. 1*B*). In FXI-deficient plasma with and without CTI (Fig. 1 *C* and *D*), thrombin generation occurred but was delayed compared to NPP and FXII-deficient plasma. PKa failed to induce thrombin generation in FIX-deficient (Fig. 1*E*) and FX-deficient (Fig. 1*F*) plasmas.

To determine whether the amount of thrombin generated by PKa in the absence of FXII was able to form fibrin, clot formation was monitored by measuring turbidity. For this purpose, clotting in NPP and plasmas deficient in FXII, FXI, FIX, and FX was triggered by recalcification and addition of increasing concentrations of PKa in the presence of PLs. The results confirmed that PKa initiates the intrinsic pathway in a dose-dependent manner in NPP (Fig. 1*G*), and triggers clot formation even in the absence of FXII or FXI (Fig. 1 *H* and *I*). Prolonged lag times were observed in FXII- and FXI-deficient plasmas (Fig. 1 *H* and *I*) compared to NPP, consistent with the less efficient thrombin generation observed in these plasmas (Fig. 1 *B*–*D*). In contrast to thrombin generation profiles showing no thrombin generation in FIX- and FX-deficient plasmas (Fig. 1 *E* and *F*), some fibrin formation was observed (Fig. 1 *J* and *K*), albeit only at high PKa concentrations. It is possible that minute amounts of thrombin are produced in FIX- and FX-deficient plasmas that are too low for detection but sufficient to initiate fibrin formation.

### PKa Directly Cleaves FIX in a Time- and Dose-Dependent Manner and Does Not Require Cofactors.

**Fig. 2.**
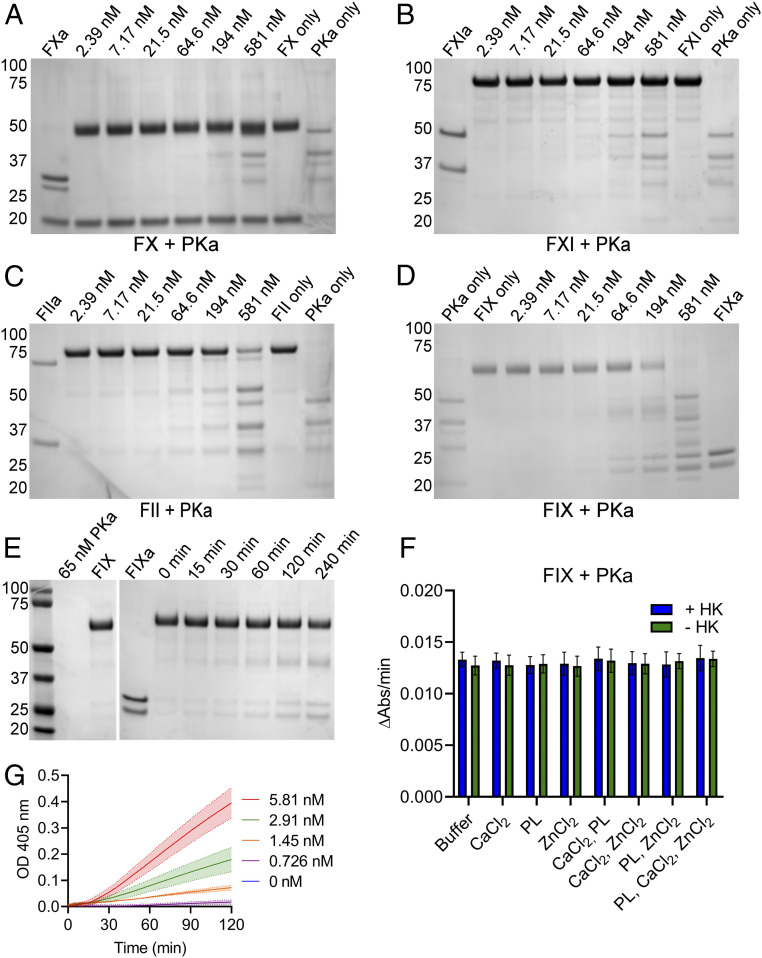
PKa can selectively cleave FIX in a dose- and time-dependent manner. (*A*–*D*) The ability of PKa to cleave zymogen FX, FXI, prothrombin (FII), and FIX was determined by incubating reaction mixtures containing 0.5 mg/mL zymogen protein with a concentration series of PKa (2.39 to 581 nM), in the presence of 1.5 mM CaCl_2_ and 10 μM PLs. Samples were incubated for 1 h at 37 °C before analysis by SDS-PAGE under reducing conditions. PKa titration incubated with FX (*A*), FXI (*B*), FII (*C*), and FIX (*D*). (*E*) The time dependency of FIX cleavage by PKa was assessed by incubating 0.5 mg/mL FIX with 65 nM PKa in the presence of 1.5 mM CaCl_2_ for up to 240 min. Samples were assessed by SDS-PAGE under reducing conditions. (*F*) 5 nM PKa and 300 nM FIX protein were mixed in the presence of a variety of buffer conditions containing PLs (10 μM), CaCl_2_ (2.5 mM), and ZnCl_2_ (10 μM). Each of these conditions was assessed in the presence (blue bars) and absence (green bars) of 12 nM HK. Cleavage of chromogenic substrate S-2765 by samples was measured for 1 h. Data represent the rate of chromogenic substrate cleavage over 1 h, mean ± SD; *n* = 3. (*G*) 300 nM FIX protein was added to 0.726 to 5.81 nM PKa in the presence of CaCl_2_ (2.5 mM) and ZnCl_2_ (10 μM), followed by the addition of chromogenic substrate S-2765. Samples containing only PKa (0.726 to 5.81 nM) were run in parallel. Data represent PKa subtracted, mean ± SD; *n* = 3. In A–E, positions of molecular mass standards (in kDa) are shown to the left of each gel image.

To determine which proteins of the coagulation cascade downstream of FXII were activated by PKa, cleavage assays were performed by incubating FX, FXI, prothrombin, or FIX with a concentration series of PKa. Cleavage products were then analyzed by reducing sodium dodecyl sulfate-polyacrylamide gel electrophoresis (SDS-PAGE). PKa did not cleave FX or FXI at any of the PKa concentrations (Fig. 2 *A* and *B*). The additional bands observed in the lanes where high concentrations of PKa were used were consistent with the migration of PKa only. In contrast, PKa cleaved prothrombin (Fig. 2*C*) and FIX (Fig. 2*D*), demonstrating cleavage of these zymogens to generate their activated enzymes. Prothrombin was cleaved at PKa concentrations ≥64.5 nM, while FIX was cleaved by concentrations ≥21.5 nM PKa.

We next investigated the generation of FIXa over time by PKa by incubating FIX (0.5 mg/mL) with PKa (65 nM) for up to 240 min in the presence of CaCl_2_. Samples were assessed by reducing SDS-PAGE and demonstrated cleavage of FIX to FIXa over time (Fig. 2*E*). Having demonstrated that PKa activation of FIX occurred in the presence of CaCl_2_, we next investigated the cofactor-dependency of this reaction in chromogenic substrate assays using purified FIX and PKa proteins. FIX and PKa proteins were first assessed for purity by mass spectrometry analysis to exclude the possibility of contaminating proteins contributing to chromogenic substrate cleavage. These analyses confirmed that FIX protein did not contain any contaminants, while PKa contained FXII (*SI Appendix*, Table S1), which was not of concern, as other groups have demonstrated that FXII is not able to cleave FIX to FIXa (26, 27). Assays were performed with combinations of PLs, CaCl_2_, and ZnCl_2_ in the presence and absence of HK to determine the effect of these combinations on FIX activation by PKa. Substrate cleavage occurred without PLs, CaCl_2_, or ZnCl_2,_ and the presence of HK did not have an accelerating effect on chromogenic substrate cleavage in samples containing FIX+PKa (Fig. 2*F*). A further experiment performed in the presence of EDTA confirmed that the activation of FIX by PKa does not require CaCl_2_ (*SI Appendix*, Fig. S1).

Rate of chromogenic substrate cleavage by FIXa increased in samples as PKa concentration increased (Fig. 2*G*), demonstrating that PKa cleaves FIX in a dose-dependent manner. In these assays, increasing concentrations of PKa were added to FIX before the immediate addition of chromogenic substrate S-2765. Samples containing only PKa were run in parallel, and the signals from these samples were used for baseline subtraction (Fig. 2*G*).

Subsequently, kinetic parameters of PKa activation of FIX were determined alongside FXIa activation of FIX to allow comparison of these two FIX activators. The *K*_m_ values for PKa and FXIa were not significantly different; the *K*_m_ for PKa activation of FIX was 154.4 nM (95% confidence interval [CI] 95.32 to 248.1 nM), and that for FXIa was 299.4 nM (95% CI, 190.6 to 477.5 nM). The kcat of PKa and FXIa were significantly different: 0.022 s^−1^ (95% CI 0.019 to 0.026 s^−1^) for PKa and 0.285 s^−1^ (95% CI, 0.244 to 0.339 s^−1^) for FXIa. The kcat/*K*_m_ for PKa and FXIa also differed significantly (Table 1), suggesting that FXIa is ∼6.5-fold more efficient as an activator of FIX (0.951/0.144 = 6.6), which is accounted for by differences in the kcat. Kinetic parameters are summarized in Table 1, and the graphs for determining rates of FIX activation by PKa and FXIa are presented in *SI Appendix*, Fig. S2. However, plasma concentrations of PK and FXI are 581 nM and 30 nM, respectively; that is, PK is 19.4-fold more abundant than FXI. Therefore, given the plasma concentration of FIX of 89 nM, we calculate that if 1% of plasma FXI were activated (0.3 nM FXIa), then the rate of FIX activation would be 0.020 nM/s, while if 1% of plasma PK were activated (5.81 nM PKa), then the rate of FIX activation by PKa would be 0.047 nM/s. Under these conditions, activation of FIX by PKa would exceed that by FXIa by 2.3-fold (rate = E_0_ × kcat × [FIX]/(*K*_m_ + [S]), where E_0_ is the concentration of PKa or FXIa and [S] is the concentration of FIX).

**Table 1.** Summary of kinetic parameters determined for the activation of FIX by PKa and FXIa

Parameter	PKa		FXIa	
	Mean	95% CI	Mean	95% CI
*V*_max_, nM/s	0.0646	0.0560–0.0749	0.0427	0.0365–0.0509
kcat , s^−1^	0.0222	0.0193–0.0258	0.2847	0.2435–0.3394
*K*_m_, μM	0.1544	0.0953–0.2481	0.2994	0.1906–0.4775
kcat/*K*_m_, μM^−1^s^−1^	0.1439	0.2021–0.1040	0.9508	1.2777–0.7108

Kinetic parameters for PKa and FXIa activation of FIX were determined in reactions in which 2.905 nM PKa and 0.15 nM FXIa were incubated with a concentration series of FIX, and cleavage of chromogenic substrate S-2765 was monitored over time. Data are the mean of data from three independent experiments, each performed in triplicate.

### Generation of FIXa by PKa Occurs in the Presence of FXII.

To determine the physiological relevance of PKa cleavage of FIX, we monitored the formation of cleavage products in assays in which PKa was present with FXII, PKa’s primary substrate. Generation of αFXIIa, βFXIIa, and FIXa was evident by PKa in a time-dependent manner (*SI Appendix*, Fig. S3).

### PKa Binds to FIX with High Affinity.

**Fig. 3.**
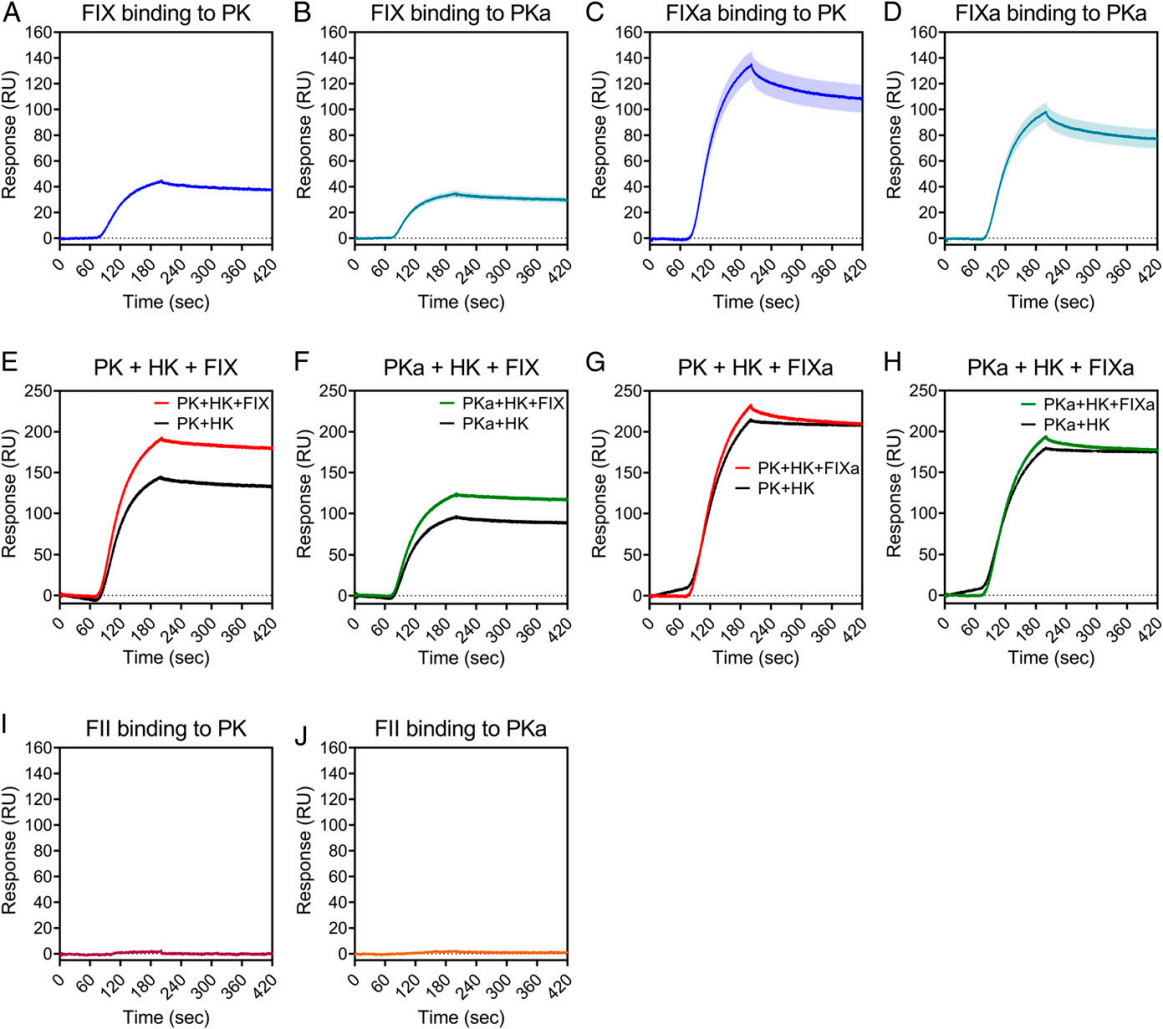
PK(a) binds to FIX(a) with high affinity. Binding kinetics and affinities of the interaction of FIX and FIXa (± HK), and prothrombin (FII) with PK and PKa were studied by SPR using a Pioneer system. FIX (*A* and *B*) and FIXa (*C* and *D*) proteins were injected over a PK- or PKa-immobilized surface. Data are mean ± SEM; *n* = 3. (*E*–*H*) Experiments were also performed in the presence of HK. FIX was preincubated with HK and flowed over a PK-immobilized (*E*) or PKa-immobilized (*F*) surface. FIXa was preincubated with HK and injected over a PK-immobilized (*G*) or PKa- immobilized (*H*) surface. (*I* and *J*) Binding of FII to PK (*I*) and PKa (*J*). *n* = 1 for experiments in *E*–*J*.

We sought to define the binding affinities involved in the interaction of PK(a) with FIX(a) using surface plasmon resonance (SPR). These experiments determined high-affinity interactions of PK and PKa for FIX (Fig. 3 *A* and *B*) and FIXa (Fig. 3 *C* and *D*). The affinity of interaction, *K*_D_, for PK binding to FIX was 2.167 ± 0.003 nM, and to FIXa was 0.492 ± 0.001 nM (*K*_D_ ± SD). The *K*_D_ for PKa binding to FIX was 1.701 ± 0.003 nM, and to FIXa was 0.700 ± 0.010 nM (Fig. 3 *A*–*D*). *K*_D_ values and rates of association (*K*_a_) and dissociation (*K*_d_) are summarized in Table 2.

**Table 2.** Summary of binding affinities and kinetics of binding to PK and PKa

Parameter	PK			PKa		
	*K*_a_ ± SD, M^−1^s^−1^	*K*_d_ ± SD, s^−1^	*K*_D_ ± SD, nM	*K*_a_ ± SD, M^−1^s^−1^	*K*_d_ ± SD, s^−1^	*K*_D_ ± SD, nM
FIX	1.103 ± 0.001e5	2.39e-4	2.167 ± 0.003	1.342 ± 0.002e5	2.28e-4	1.701 ± 0.003
FIXa	1.311 ± 0.001e5	6.45 ± 0.02e-5	0.492 ± 0.001	1.341 ± 0.005e5	9.30 ± 0.1e-5	0.700 ± 0.010
HK	1.520 ± 0.004e5	3.73 ± 0.01e-5	0.245 ± 0.001	1.037 ± 0.004e5	2.82e-5	0.272 ± 0.001
FII	No binding			No binding		

The binding of FIX, FIXa, HK, and FII to PK and PKa was investigated by SPR, and affinity (*K*_D_) and rate constants for association (*K*_a_) and dissociation (*K*_d_) were determined.

The high-affinity interaction of PK with FIX and FIXa prompted the question of whether binding would occur in circulation, considering that PK circulates predominantly (∼70 to 80%) in a 1:1 noncovalent complex with HK (29). SPR studies showed a high-affinity interaction of PK and PKa for HK (Table 2). FIX was able to form a complex with PK in the presence of HK, as indicated by the increase in resonance units (RU) in the PK+HK+FIX sample compared with the PK+HK sample (Fig. 3*E*), which was approximately equal to the signal from FIX+PK binding (Fig. 3*A*). Similarly, SPR data suggested that PKa, FIX, and HK were able to form a complex (Fig. 3*F*). In contrast to FIX, FIXa contributed no more binding to PK or PKa when HK was present (Fig. 3 *G* and *H*), suggesting that FIXa does not form a complex with PK(a) and HK. To summarize, these data show that PK+HK+FIX and PKa+HK+FIX complexes can exist, but not when FIX is activated; that is, PK+HK+FIXa and PKa+HK+FIXa complexes cannot exist.

We next investigated the interaction of PK and PKa with prothrombin, as our data demonstrated cleavage of prothrombin by PKa (Fig. 2*C*). No binding of PK or PKa to prothrombin was detected (Fig. 3 *I* and *J* and Table 2).

## Discussion

Despite early studies demonstrating an ability of PKa to cleave FIX (23, 24, 30), the general dogma for many years was that PKa’s role in coagulation was solely FXII-dependent, ultimately resulting in thrombin generation via FXIa generation. Puy et al. (26) showed the possible potential for PKa to contribute to clot formation in a manner independent of FXI, by demonstrating that long-chain polyP-activated FXII could generate PKa, which subsequently activated FIX. More recently, Visser et al. (27) and Noubouossie et al. (28) have provided indirect evidence through the measurement of FIXa-antithrombin complexes that PKa can activate FIX in an FXI-independent manner. In this report, we provide direct evidence of high-affinity binding interactions between PK(a) and FIX(a). We also demonstrate time-and dose-dependent activation of FIX by PKa that ultimately causes thrombin generation and results in fibrin formation in an FXI-independent manner. Furthermore, we determined kinetic parameters of FIX activation by both PKa and FXIa, allowing direct comparison of the two FIX activators under the same experimental conditions.

To confirm that PKa generates thrombin independent of its activity on FXII, we first measured thrombin generation in FXII- and FXI-deficient plasmas triggered with a concentration series of PKa. As expected, PKa was most efficient at generating thrombin when FXII was present in NPP. However, in the absence of FXII or FXI, it was still possible to observe dose-dependent thrombin generation. The addition of CTI did not affect thrombin generation in the FXII- or FXI-deficient plasmas, indicating that the thrombin generated was not FXII-dependent. When PKa was added to FIX- or FX-deficient plasmas, no thrombin was generated, suggesting that FIX and FX are required to generate detectable levels of thrombin. These findings were confirmed by Noubouossie et al. (28), who demonstrated that purified PKa initiated thrombin generation in NPP and FXII- and FXI-deficient plasmas, but not in FIX-deficient plasma. To expand on this, we performed experiments to characterize the role of PKa on clot formation using turbidity analyses. Using FXII- or FXI-deficient plasma, PKa was able to induce clot formation, albeit with lower efficiency at the lower concentrations of PKa. In addition, we showed that despite the absence of thrombin generation in FIX- and FX-deficient plasmas, there was fibrin formation at the highest PKa concentrations. Since FXIa has been shown to activate FX (26) and induce thrombin generation in FIX-deficient plasma by activating FV and FX (31), it is possible that minute amounts of thrombin are produced in FIX-deficient plasma by these mechanisms, which are not detectable by thrombin generation but are sufficient for fibrin formation. Fibrin formation in FX-deficient plasma at the highest PKa concentration may be a result of direct cleavage of prothrombin by PKa, as has been observed by others (26, 32, 33). However, we do not anticipate that this occurs physiologically. Together, the thrombin generation and turbidity experiments confirm that PKa can act independently of FXII and FXI to result in the generation of thrombin and in fibrin formation.

Having demonstrated that PKa could initiate clotting in the absence of FXII and FXI, we next sought to determine which zymogens of the coagulation cascade downstream of FXII could be cleaved when incubated with increasing concentrations of PKa. PKa cleaved FIX to FIXa and prothrombin to thrombin, albeit at higher PKa concentrations than were required for cleavage of FIX. Given that higher concentrations of PKa were required to cleave prothrombin, it is likely that this process has relatively less physiological significance than PKa activation of FIX. Furthermore, we did not detect any binding of PK or PKa to prothrombin in our SPR experiments, suggesting that PKa cleavage of prothrombin is an inefficient reaction occurring only in the presence of a high PKa concentration. At high concentrations, PKa has trypsin-like qualities, which would allow cleavage of prothrombin by PKa despite the low affinity of the proteins for each other.

In contrast to the absence of PKa binding to prothrombin in SPR studies, we demonstrated high-affinity binding interactions between PK(a) and FIX(a), with binding affinities in the low nanomolar range. FIX circulates in plasma at ∼89 nM (24), and thus the interaction with PK and PKa is likely to occur under physiological conditions and potentially play a role in thrombin generation and clot formation under appropriate conditions. Puy et al. (26) showed that this route is likely to be relevant in the context of a surface such as long-chain polyP during bacterial infections, and Visser et al. (27) demonstrated that it occurs in vivo in FXI-deficient mice following treatment with long-chain polyP. It is plausible that other surfaces may also drive this role of PKa activity toward FIX in physiological circumstances. Further SPR experiments with HK indicated complex formation between PK(a), HK, and FIX, but not FIXa. These data suggest that PK, which circulates with HK (29), can bind FIX, and that once activation occurs, active FIXa no longer binds. It is necessary to consider the limitations of the SPR system when interpreting these data. Although we detected an increased binding signal suggesting formation of a complex between PK(a), HK, and FIX, due to the unlabeled nature of the SPR system, it is not possible to say with complete certainty that FIX was not binding to HK in these experiments. However, to our knowledge, there have been no reports of FIX-HK binding, and thus it is much more likely that we detected FIX binding to PK(a) in the presence of HK.

Subsequently, we investigated the activation of FIX by PKa by direct measurement of FIXa generation in chromogenic substrate assays. To be confident in the interpretation of substrate cleavage assays using purified FIX and PKa, we submitted samples of these proteins for mass spectrometry analysis to identify any possible contaminants that could have contributed to substrate cleavage. We found that the PKa sample was contaminated with FXII, while FIX protein was free from contamination. The presence of FXII in PKa samples could not have affected chromogenic substrate cleavage (by activating FIX in place of/in addition to PKa), as others have shown that FXII(a) cannot activate FIX directly (26, 27). Our data indicate that the activation of FIX by PKa occurs in the absence of CaCl_2_, supporting the conclusion reached in the earlier studies of Osterud et al. (23, 24) and Puy et al. (26). Additionally, our data demonstrate that this reaction can occur in the absence of ZnCl_2_ and PLs, with vesicles containing 60% 1,2-dioleoyl-sn-glycero-3-phosphocholine (DOPC), 20% 1,2-dioleoylsn-glycero-3-phosphoethanolamine (DOPE), and 20% 1,2-dioleoyl-sn-glycero-3-phosphoserine (DOPS), and, further, that the presence of HK does not have any effect on the reaction. In a study of the activation of FXII by PKa, Wang et al. (34) reported a requirement of HK for PKa; however, our data suggest that HK has little effect on the FIX-activating activity of PKa. In summary, our data show that the activation of FIX by PKa can happen in the absence of a surface or in the presence of PLs, and that HK plays no role as an accelerating protein cofactor under these conditions. However, given the SPR data showing formation of a trimolecular complex of PK(a), HK, and FIX, it may be that HK has a role in FIX activation in the presence of other surfaces, such as long-chain polyP (35, 36).

A more detailed analysis of the kinetic characteristics of PKa activation of FIX, and comparison with FXIa activation of FIX revealed that PKa may contribute significantly to FIXa generation under physiological conditions. The catalytic efficacy of FXIa activation of FIX was ∼6.5-fold more efficient than the PKa activation of FIX. However, given that the plasma concentration of PK is ∼19.4 times more abundant than FXI, if similar proportionate amounts of PKa and FXIa are generated with respect to percentage of each zymogen activated, then according to the formula rate = E_0_ × kcat × [FIX]/(*K*_m_ + [S]) (where E_0_ is the concentration of PKa or FXIa and [S] is the concentration of FIX), PKa is ∼2.3 fold more likely than FXIa to result in FIX activation, under our experimental conditions. It remains to be determined if other surfaces/proteins influence these reactions.

PK has close sequence homology with FXI; both genes evolved from a common ancestor, and the proteins share ∼58% sequence homology (37). Analogous to FXI, PK is composed of four apple domains that form the heavy chain of PK and a C-terminal protease domain (38). Given the similarity between FXI and PK, it is perhaps not surprising that both proteins can activate FIX. A study by Sun and Gailani (25), in which recombinant FXI proteins were expressed with each of the four apple domains of the heavy chain individually replaced with the corresponding domain from the homologous PK, suggested that FXIa could be ∼45 times more efficient than PKa as an ex vivo activator of FIX; however, that study did not compare WT PK with FXI proteins directly. Others have found larger differences in the FIX-activating abilities of FXIa and PKa. Osterud et al. (24) reported that FXIa was ∼20,000 times more active than PKa at activating FIX in their experimental setup. However, that early study was based on subsampling of a single set of experimental reaction conditions for FXIa or PKa with FIX, in contrast to the detailed kinetic analysis that we have performed. More recently, Visser et al. (27) demonstrated that inhibition of PKa did not inhibit FIXa generation in normal plasma, compared with a large decrease in the detection of FIXa in FXI-deficient plasma. Our data show that FXIa is ∼6.5 times more efficient than PKa as an activator of FIX, which is largely due to the significantly higher kcat of FXIa. However, in the context of the greater PK plasma concentrations compared to FXI, it is plausible that PKa contributes significantly to the overall generation of FIXa.

These data suggest that PKa is a more versatile protease than originally suspected in the context of hemostasis and thrombosis. PKa contributes to thrombin generation primarily through activation of FXII, although we have demonstrated that PKa activation of FIX can occur independently of FXII and FXI. It remains to be determined under what conditions PKa activates FIX over FXII, or whether these activation events occur concurrently physiologically. It seems plausible that this event is physiological, given the tight binding interaction between PK(a) and FIX(a). PK could potentially be a carrier protein for FIX, bringing FIX and PKa in close proximity to a surface where clot formation is likely to occur. The implications of PKa activation of FIX are relevant to the development of new anticoagulants targeting FXI and FXII (39, 40).

**Fig. 4.**
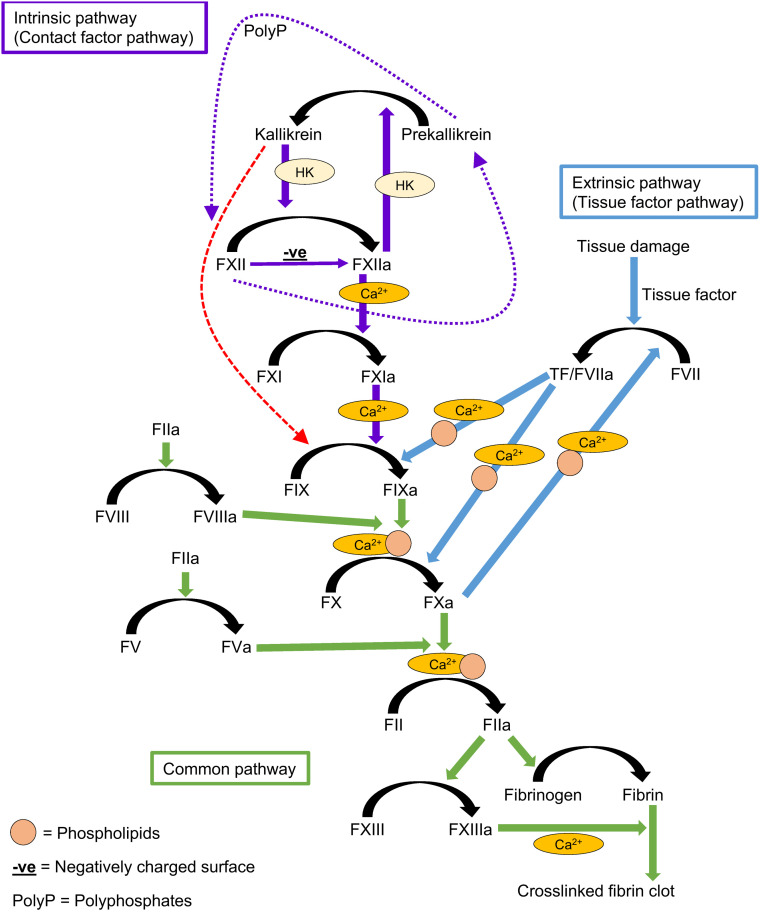
The coagulation cascade. The intrinsic pathway is initiated after activation of FXII by contact with a negatively charged surface. FXIIa can activate PK to PKa, and PKa can activate more FXII. In the presence of a surface, reciprocal activation of FXII and PK to FXIIa and PKa is amplified by cofactor HK. FXII has intrinsic proteolytic activity that is able to activate PK without a surface (purple dotted arrows). Similarly, PK has proteolytic activity that can activate FXII, but this reaction requires a surface (purple dotted arrows). Following FXIIa activation, FXI is activated to FXIa, and FIX is activated to FIXa. The extrinsic pathway is initiated following tissue damage and exposure of tissue factor. Both pathways converge at the activation of FX to FXa and conversion of prothrombin to thrombin. Thrombin cleaves fibrinogen to fibrin, which polymerizes to form fibrin fibers and also activates FXIII, which introduces cross-links into the fibrin network, stabilizing it. Many reactions of the coagulation cascade require calcium (Ca^2+^) or PLs (yellow circles). We and others have demonstrated that PKa can directly activate FIX, bypassing FXI (red dashed arrow). Zymogens are indicated by roman numerals, and their activated forms end with an “a”.

It has previously been considered that FXIa inhibitors would target all the actions of FXIIa. However, the mechanism of FIX activation by PKa bypasses FXI. Therefore, it is possible that FXIIa inhibitors may contribute to reduced PKa generation, which in turn would lead to reduced FIX activation and subsequent thrombin generation, independent of FXIa activity. To summarize, it is highly plausible that PKa activation of FIX is a mechanism responsible for thrombin generation and fibrin formation, and it needs to be considered in a revised model of the coagulation cascade (Fig. 4).

## Materials and Methods

### Chemicals.

PKa, PK, HK, and CTI were obtained from Enzyme Research Laboratories. Prothrombin, FIX, FX, FXI, and FXII were purchased from Haematologic Industries. Thrombin calibrator and FluCa kit (buffer and substrate) were purchased from Stago. FIX-, FX-, FXI-, and FXII-deficient plasmas were obtained from George King Bio-Medical. CaCl_2_, ZnCl_2_, NaCl, 4-(2-hydroxyethyl)-1-piperazineethanesulfonic acid (Hepes), bovine serum albumin, and InstantBlue gel stain were purchased from Sigma-Aldrich. NuPAGE 4–12% Bis-Tris Protein Gels, MES SDS running buffer, LDS sample buffer, and sample reducing agent were obtained from Thermo Fisher Scientific. Precision Plus Protein Dual Color Standards was obtained from Bio-Rad. Chromogenic substrate S-2765 was obtained from Quadratech Diagnostics. The synthetic PLs DOPC, DOPS, and DOPE were purchased from Avanti Polar Lipids. Unless stated otherwise, the buffer used was Hepes-buffered saline (HBS) containing 20 mM Hepes and 137 mM NaCl, pH 7.4.

### PL Preparation.

PL vesicles were composed of 60% DOPC, 20% DOPS, and 20% DOPE and were prepared as described previously (41). In brief, PLs were dried under nitrogen gas, resuspended in HBS, vortexed, and extruded through a 1-μm membrane.

### NPP Samples.

Whole blood was collected from 30 healthy volunteers into 0.109 M trisodium citrate-treated tubes. Informed written consent was obtained from each volunteer in accordance with the Declaration of Helsinki. Ethical approval was obtained from the University of Leeds Medical School Ethical Committee (HSLTLM/12/045). Free flow or minimal suction was used during blood sampling. Blood samples were centrifuged twice at room temperature: 30 min at 3,000 × *g*, followed by 10 min at 11,000 × *g*. Platelet-poor plasma supernatants were transferred into clean polypropylene tubes using Pasteur pipettes. Pooled plasma was divided into 0.5-mL aliquots, snap-frozen in liquid nitrogen, and stored at −80 °C.

### Thrombin Generation.

Thrombin generation was measured using a Thrombinoscope (Stago) and performed in Immulon 2HB, round-bottom 96-well plates (Thermo Fisher Scientific). In brief, 60 μL of each plasma sample was spiked with 2.39 to 581 nM PKa in 20 μL with 10 μM PLs. Samples containing 20 μL of thrombin calibrator (Diagnostica Stago) were run in parallel with each cycle of test sample. Thrombin generation was triggered by the addition of PKa, followed by 20 μL of calcium–fluorogenic substrate reagent (16.7 mM calcium and 0.42 mM substrate final reaction concentrations). In some experiments, CTI (1.6 μM) was added to the plasma samples to block FXII activity. All concentrations are final. Thrombograms were produced by monitoring fluorescence at 37 °C at 20-s intervals for 120 min. Each set of conditions was tested in triplicate.

### Turbidity Measurements of Fibrin Formation.

Fibrin polymerization in plasma samples was measured in clear flat bottom polystyrene 96-well plates (Thermo Fisher Scientific) by monitoring turbidity at 340 nm every 12 s for 1 h using a PowerWave HT Microplate Spectrophotometer (BioTek). Citrated plasma solutions (25% plasma) were added to a 96-well polystyrene plate (Thermo Fisher Scientific), and gelation was initiated with CaCl_2_ (10 mM) in combination with a range of PKa concentrations (0.797 to 581 nM). Experiments were performed in the presence of 10 μM PL vesicles (all concentrations are final). Each set of conditions was tested in triplicate.

### Zymogen Cleavage by PKa, Assessed by SDS-PAGE.

Samples of zymogen (FX, FXI, prothrombin, or FIX; 0.5 mg/mL), PKa (2.39 to 581 nM), 10 μM PLs, and 1.5 mM CaCl_2_ were incubated at 37 °C (all concentrations are final). The addition of reducing agent and sample buffer to samples stopped the reactions. Samples were boiled at 95 °C for 10 min before loading. Gels were run for 50 min at 200 V and stained overnight using InstantBlue gel stain and then imaged on Syngene G:BOX Chemi and GeneSys software.

### Cleavage of FIX by PKa in the Presence of FXII.

Here 6 μM zymogen FIX and FXII were incubated with 100 nM PKa in the presence of 10 μM PLs, 1.5 mM CaCl_2,_ 10 μM ZnCl_2_, and 100 nM HK at 37 °C for up to 120 min (all concentrations are final). Reactions were stopped by the addition of reducing agent and boiling for 10 min at 95 °C, and samples were assessed by SDS-PAGE under reducing conditions. Band intensities on the stained gel were determined using ImageJ.

### Activation of Factor IX by Plasma PKa in Chromogenic Assays.

FIX and PKa proteins were assessed for purity by mass spectrometry analysis at the Biomolecular Mass Spectrometry Facility of the University of Leeds (*SI Appendix*). The activation of zymogen FIX by PKa was assayed in clear polystyrene 96-well flat-bottom plates (Thermo Fisher Scientific) with 300 nM FIX and 5 nM PKa. Conditions tested included the addition of purified HK (12 nM), PLs (10 μM), CaCl_2_ (2.5 mM), and ZnCl_2_ (10 μM). The generation of FIXa was determined by monitoring cleavage of S-2765 (5 mM) using a PowerWave HT Microplate Spectrophotometer (BioTek) at 405 nm every 12 s for 1 h. For PKa dose–response experiments, a concentration curve of PKa (0.726 to 5.81 nM) was added to the plate, with and without FIX (300 nM) with CaCl_2_ (2.5 mM) and ZnCl_2_ (10 μM). Cleavage of chromogenic substrate S-2765 (5 mM) was measured as described above. For experiments with EDTA, FIX (300 nM) and PKa (5 nM) were added to wells containing CaCl_2_ (1.5 mM) or EDTA (1 mM). Chromogenic substrate S-2765 was added, and substrate cleavage was measured as described above.

### Determination of Kinetic Parameters.

PKa (2.905 nM) and FXIa (0.15 nM) were added to a concentration series of FIX (37.5 to 1,200 nM) before the addition of 5 mM S-2765, in the presence of 2.5 mM CaCl_2_ and 10 μM ZnCl_2_ (all final concentrations). The generation of FIXa was determined by monitoring the cleavage of S-2765 as described above. Rates of FIX activation by PKa and FXIa (in pM/s) were determined using a Shiny app for calculating zymogen activation rates (version 0.62) (42). This app uses predetermined parameters for the action of FIXa on the chromogenic substrate S-2765 to calculate the initial rate of FIXa generation in pM/s from reaction time courses using slopes of absorbance vs. time squared. The values for *K*_m_ (40.46 mM) and kcat (2.43 s^−1^) for FIXa on S-2765 were not well estimated from fitting of Michaelis–Menten curves (GraphPad Prism version 8) because the *K*_m_ was so high. However, when rates were measured at [S] << *K*_m_, the individual parameters of kcat and *K*_m_ were not important; only their ratio was important, and this could be estimated from rate ∼ [Eo]⋅[S]⋅kcat/*K*_m_, which is linear where [S] << *K*_m_. Therefore, linear fits for initial rates of time squared plots were performed over the first 20 min of the reaction, and a 60-s delay was included for the time between reaction mixing and the first reading.

### SPR.

SPR was performed using the Pall/ForteBio Pioneer biosensor platform (Molecular Devices) at a temperature of 22 °C. More details are provided in *SI Appendix*. Assay running buffer (RB), containing 10 mM Hepes, 140 mM NaCl, 1.5 mM CaCl_2_, and 40 µM ZnCl_2_ pH 7.4, was filter-sterilized and degassed. A COOHV chip was installed into the Pioneer platform and primed three times using RB. The surface was preconditioned for amine coupling, and proteins were immobilized to the sensor surface using a standard amine coupling method (43). Phenylmethylsulfonyl fluoride (PMSF)-treated PK was immobilized to flow cell (FC)-1, and PKa was immobilized to FC-3. Remaining active NHS-ester bonds on FC-1, FC-2, and FC-3 were blocked by injection of 1 M ethanolamine (pH 8.5) at 20 µL/min for 5 min. Final immobilization levels of PK and PKa were engineered to provide a theoretic binding maximum (R_MAX_) of 100 to 200 RU. Three prime functions were performed before analysis using RB. All analytes were diluted to 250 nM in RB and injected over the sensor surface using a OneStep 100% loop injection, with Taylor dispersion to create a concentration gradient through the capillary tube before entering the FC, at a flow rate of 30 µL/min with a dissociation time of 600 s. Chip surface regeneration following binding was performed with bursts of regeneration mixture (50 mM EDTA pH 7.35, 600 mM imidazole pH 7.4, 6 mM NaAc pH 5.0, and 5% vol/vol HCl), followed by RB. Experiments with HK were performed using a similar methodology, as described in detail in *SI Appendix*. In brief, analytes were diluted to 250 nM into one vessel to permit preincubation with 250 nM HK prior to injection.

Data were collected with the SPR program (Molecular Devices). Analyte molecular weights were input to calculate the diffusion coefficient (m^2^/s), and the target ligand molecular weights and surface density were input to calculate R_MAX_. Cycle data were aligned to the start of injection, and the channel-channel alignment box was checked. Data were analyzed with the Q_DAT_ program (Molecular Devices). Each FC was zeroed to the RU prior to analyte titration. Reference curves (FC-2) were subtracted, and the data were blanked to the closest buffer blank. The kinetics and affinity of binding interactions were obtained by fitting a simple ka/kd 1 or 2 binding site model. The assay was run in triplicate, and the SEM was calculated using GraphPad Prism 8 software.

## Supplementary Material

Supplementary File

## Data Availability

All study data are included in the article and supporting information.

## Change History

January 28, 2021: The license for this article has been updated.
