# Supplementary material for: Kallikrein directly interacts with and activates Factor IX, resulting in thrombin generation and fibrin formation independent of Factor XI
Source: Proc Natl Acad Sci U S A. 2021 Jan 28;118(3):e2014810118. doi: 10.1073/pnas.2014810118 (PMC7826336; doi:10.1073/pnas.2014810118)
Supplement: Supplementary File [file pnas.2014810118.sapp.pdf]

Supplementary Information for

Kallikrein directly interacts with and activates Factor IX, resulting in thrombin generation and fibrin formation independent of Factor XI

Katherine J. Kearney, Juliet Butler, Olga M. Posada, Clare Wilson, Samantha Heal, Majid Ali, Lewis Hardy, Josefin Ahnström, David Gailani, Richard Foster, Emma Hethershaw, Colin Longstaff, Helen Philippou

Correspondence: Professor Helen Philippou, Leeds Institute of Cardiovascular and Metabolic Medicine, University of Leeds, Leeds, UK, LS2 9JT.
Email: H.Philippou@leeds.ac.uk. Phone: +44(0)113 343 7768.

This PDF file includes:

Table S1
Supplementary data figures S1-S3
Supplementary methods
SI references

Supplementary Data

Mass spectrometry analysis of FIX and PKa proteins

To ensure that FIX and kallikrein (PKa) protein samples did not contain any contaminants which could have affected chromogenic substrate cleavage in purified protein assays, protein samples were submitted for analysis to the Biomolecular Mass Spectrometry Facility at The University of Leeds. Table S1 shows that PKa sample contained PKa and FXII, while FIX protein sample did not contain any contaminants.

**Sample submitted**	**Number of peptides detected**	**% Sequence coverage**	**Average mass**	**Protein identification**
PKa	125	82	71370	Plasma kallikrein
	8	21	67792	Factor XII
Factor IX	54	63	51778	Factor IX

Table S1. Results of mass spectrometry analysis of PKa and FIX protein samples.

Supplementary Figures

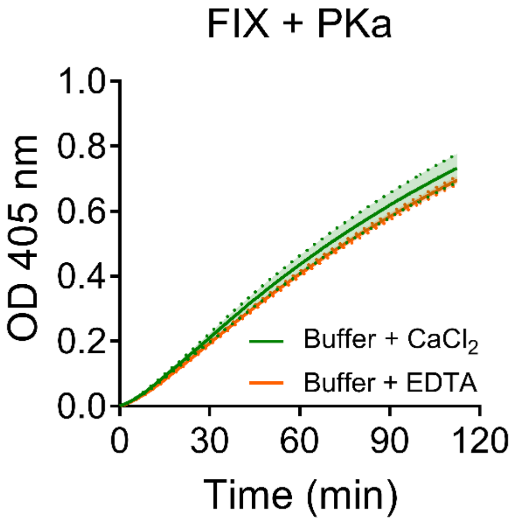

Fig. S1. Activation of FIX by PKa occurs in the presence of EDTA. 5 nM PKa and 300 nM FIX were added to wells containing buffer and either 1.5 mM $CaCl_2$ or 1 mM EDTA. Chromogenic substrate S-2765 (1 mM) was added and its cleavage monitored over time. Data presented is chromogenic substrate cleavage, mean ± SD, n=3.

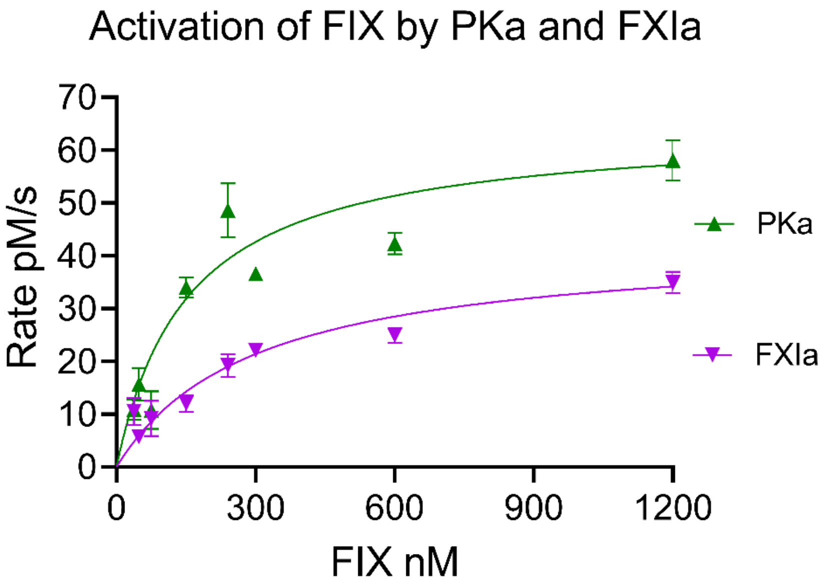

Fig. S2. Determination of kinetic parameters for FIX activation by PKa and FXIa. FIX activation by PKa (2.905 nM) and FXIa (0.15 nM) was assessed by monitoring cleavage of chromogenic substrate S-2765 (5 mM). Kinetic experiments were performed in the presence of 2.5 mM $CaCl_2$ and 10 µM $ZnCl_2$. Wells containing only PKa or FXIa were run in parallel to allow for subtraction of data coming from cleavage of the chromogenic substrate by these proteins. Rates of chromogenic substrate cleavage by generated FIXa were converted to pM/s using a Shiny App for calculating zymogen activation rates, version 0.62 [1]. Data shown is mean ± SEM of data from 3 independent experiments, each performed in triplicate.

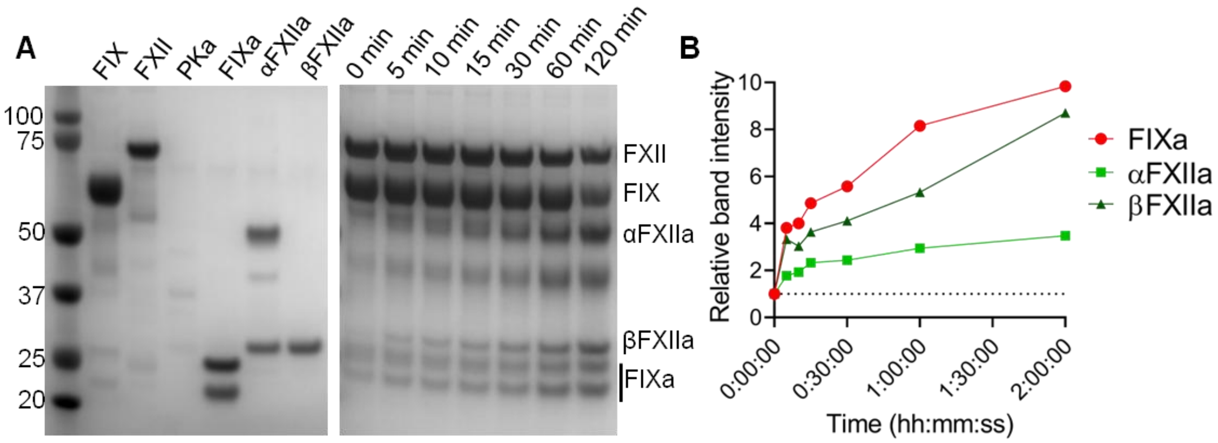

Fig. S3. Cleavage of FIX by PKa occurs in the presence of FXII. 6 μM FIX and 6 μM FXII were incubated with 100 nM PKa in the presence of 10 μM phospholipids, 1.5 mM $CaCl_2$, 10 μM $ZnCl_2$ and 100 nM HK at 37 °C for up to 120 min. Reactions were stopped by the addition of reducing agent and boiling for 10 minutes at 95 °C and samples assessed by SDS-PAGE under reducing conditions. Positions of molecular mass standards in kDa are shown to the left of the image. (B) Band volume of active FIXa, αFXIIa and βFXIIa in (A) was taken in each lane using ImageJ. Band volumes of FIXa, αFXIIa and βFXIIa at time zero were normalised to 1 and increases in relative intensity were calculated over time for the generation of FIXa, αFXIIa and βFXIIa bands.

Supplementary Methods

Protein Identification by Mass Spectrometry

Proteins were fully solubilised by adding 50 µl of 10% SDS solution to 50 µl sample. Reduction and alkylation were then performed. Dithiothreitol (DTT) was added to a final concentration of 20 mM before heating to 56 °C for 15 min with shaking. The sample was left to cool then iodoacetamide was added to a final concentration of 40 mM, before heating to 20 °C for 15 min with shaking in the dark. Proteins were further denatured by acidification, phosphoric acid was added to a final concentration of 1.2%. This step ensures destruction of all enzymatic activity and maximises sensitivity to proteolysis. Samples were then diluted with S-Trap binding buffer (100 mM triethylamonium bicarbonate (TEAB) pH 7.1 in methanol), 1 µg of trypsin reconstituted in 50 mM TEAB was added before quickly being loaded onto the S-trap column. Proteins were captured within the submicron pores of the three-dimensional trap. Proteins captured within the trap present exceptionally high surface area allowing them to be washed free of contaminants. The S-trap was washed by adding 150 µl binding buffer before being spun at 4000 g for 30 seconds. 30 µl of 0.02 µg/µl trypsin was then added to the top of the S-trap. Confinement of the protein and protease within the pores of the trap forces fast digestion as the protease is either digesting the substrate or is reflected off the sidewalls straight back to the protein to digest. S-traps were loosely capped and placed in a 1.5 ml eppendorf tube and heated to 46 °C for 15 min with no shaking. Digested peptides were eluted by first spinning the S-trap at 4000 g for 1 min. Further elutions used 40 µl 50 mM TEAB, 40 µl 0.2% formic acid, and 30 µl 50% acetonitrile with 0.2% formic acid prior to centrifugation. Elutions were combined then dried down prior to resuspension in 0.2% formic acid.

3 µl sample was injected onto an in house-packed 20 cm capillary column (inner diameter 75 µm, 3.5 µm Kromasil C18 media). An EasyLC nano liquid chromatography system was used to apply a gradient of 4–40% acetonitrile in 0.1% formic acid over 30 min at a flow rate of 250 nl/min. Total acquisition time was 60 min including column wash and re-equilibration. Separated peptides were eluted directly from the column and sprayed into an Orbitrap Velos Mass Spectrometer (ThermoFisher Scientific, Hemel Hempstead, UK) using an electrospray capillary voltage of 2.7 kV. Precursor ion scans were acquired in the Orbitrap with resolution of 60000. Up to 20 ions per precursor scan were selected for fragmentation in the ion-trap. Dynamic exclusion of 30 s was used. Peptide MS/MS data were processed with PEAKS Studio X+ (Bioinformatic Solutions Inc, Waterloo, Ontario, Canada) and searched against the Uniprot Human database (release 2019_12). Carbamiodomethylation was selected as a fixed modification, variable modifications were set for oxidation of methionine and deamidation of glutamine and asparagine. MS mass tolerance was 5 ppm, and fragment ion mass tolerance was 0.3 Da. The peptide false discovery rate was set to 1%.

Surface Plasmon Resonance

Amine Coupling Based Ligand Immobilisation

SPR was performed using the Pall/ForteBio Pioneer biosensor platform (Molecular Devices, LLC, San Jose, CA). Amine coupling was performed using the Biacore™ Amine Coupling Kit from GE Healthcare Life Sciences (GE Healthcare, Little Chalfont, UK). A COOHV chip (PS17) was installed into the Pioneer platform per the manufacturer's instructions and the prime function was performed three times using filter-sterilised (0.2 µm), degassed running buffer (10 mM Hepes, 140 mM NaCl, 1.5 mM $CaCl_2$, 40 µM $ZnCl_2$ pH 7.4). The chip surface was preconditioned for adsorption by injecting 2 bursts each of 10 mM HCl, then 50 mM

NaOH and finally 0.1% SDS at 100 µl/min for 10 seconds each. The chip was primed a further 3 times using running buffer. The total internal reflection was normalised with HPLC-grade 100% DMSO, followed by 3 prime steps with running buffer. Flow cells (FC) 1, 2 and 3 were activated by injecting a 50:50 solution of EDC/NHS at 25 µl/min for 4 minutes. PMSF-treated PK was diluted 20-fold into 10 mM Hepes pH 7.0 (pI of PK ~ 8.57) to 36 µg/ml, and was immobilised to FC-1 at a flow rate of 5 µl/minute. PKa was diluted 20-fold into 10 mM Hepes pH 7.0 (pI of PKa ~ 8.57) to 50 µg/ml and immobilised to FC-3 at a flow rate of 5 µl/minute. Remaining active NHS - ester bonds on FC-1, 2 and 3 were blocked by injection of 1 M ethanolamine pH 8.5 using FC direction 3-2-1 at 20 µl/min for 5 minutes. Final immobilisation levels of PK and PKa were engineered to provide a theoretic binding maximum (R_{MAX}) of between 100 and 200 resonance units (RU) in accordance with equation 1:

$$R_{MAX} = \frac{Mw\ (Analyte)}{Mw\ (Ligand)} \times R_L \times S_M$$

Equation 1 where:
R_{MAX} = the maximum binding capacity assigned between the immobilised ligand and the analyte (RU).
Mw Analyte = molecular mass of the molecule in solution in Daltons.
Mw Ligand = molecular mass of the immobilised ligand in Daltons.
R_L = immobilised ligand (RU).
S_M = predicted molecular stoichiometry of analyte-to-ligand.

Protein-Protein Interaction

All kinetic binding assays were performed with both the analysis and sample rack temperatures at 22 °C and data sampling set to 10 Hz. All analytes were dialysed into running buffer to a factor of 1 in 10,000,000. Analyte concentration was assessed by nanodrop spectroscopy using E1% values following dialysis. Multiple rounds of analyte trial injections were performed to assess avidity and affinity to deduce maximum analyte concentration for Taylor dispersion injections, and to deduce ideal dissociation times and regeneration conditions.

Three prime functions were performed prior to analysis using running buffer. All analytes were diluted to 250 nM using the same batch of running buffer for the blanks, assay running buffer and the OneStep® titration function. Analytes were injected into the sensor chamber at a flow-rate of 30 µl/min using the OneStep® titration function, with a loop-inject of 100% following 8 leadoff blanks and 3 bulk standard injects of 3% sucrose in running buffer for n=3 using group replicates. All analytes were allowed to dissociate for 600 seconds each. The regeneration conditions were changed according to analyte affinity and avidity. Chip surface regeneration following zymogen binding was performed by a 16 µl burst of regeneration cocktail (50 mM EDTA pH 7.35, 600 mM imidazole pH 7.4, 6 mM NaAc pH 5.0 and 5% v/v HCl) at 100 µl/min flow rate, followed by a 50 µl burst of running buffer at 100 µl/min flow rate. Chip surface regeneration following enzyme binding was performed by a 16 µl burst of regeneration cocktail at 100 µl/min flow rate, followed by a 25 µl burst of running buffer at 100 µl/min flow rate. One periodic blank was performed every 2 cycles with no regeneration. All tubing was flushed and the system purged between each run, with a 60 second wait time between each run.

HK-Complex Protein-Protein Interactions

Complex SPR experimentation followed the same methodology as those given for the protein-protein interaction with minor changes to the titration of analytes, dissociation time length and regeneration phase. All data were performed with the same sensor chip to minimise batch and immobilisation differences for optimum data quality. Analytes were diluted into the same batch of running buffer as the blanks, assay running buffer and the OneStep® titration function. Analytes were diluted to 250 nM into one vessel to permit pre-incubation with 250 nM HK prior to injection. No chip surface priming was performed with HK prior to analyte-HK inject. Analyte-HK was injected with 100% loop inject, interspersed with periodic negative control injects of HK, following 8 leadoff blanks and periodic running buffer blanks every 2 cycles. Analytes were titrated at 30 µl/min with a 1200 second dissociation time. The chip surface with regenerated with two sequential 25 µl bursts of regeneration cocktail at 100 µl/min flow rate. All tubing was flushed after each cycle, interspersed by a 60-second wait time and purged before the next cycle.

SPR using Taylor dispersion injections

SPR was performed using a OneStep® titration function based on Taylor dispersion injection (TDi) theory [2, 3]. Compared to traditional (fixed concentration) injections that record analyte binding with respect to injection time of a series of samples of different concentrations, TDi analyses a continuous analyte concentration gradient formed after a single injection in a capillary tube before the sample enters the SPR detector. This approach encodes a second, independent time domain into an analyte gradient, which permits the use of a single binding curve that does not need to reach steady-state to obtain binding affinities.

SI References

1. Longstaff, C., Development of Shiny app tools to simplify and standardize the analysis of hemostasis assay data: communication from the SSC of the ISTH. *J Thromb Haemost*. 15(5): p. 1044-1046 (2017).
2. Quinn, J.G., Modeling Taylor dispersion injections: determination of kinetic/affinity interaction constants and diffusion coefficients in label-free biosensing. *Anal Biochem*. 421(2): p. 391-400 (2012).
3. Quinn, J.G., Evaluation of Taylor dispersion injections: determining kinetic/affinity interaction constants and diffusion coefficients in label-free biosensing. *Anal Biochem*. 421(2): p. 401-10 (2012).
